# Periodontal and systemic health of morbidly obese patients eligible for bariatric surgery: a cross-sectional study

**DOI:** 10.1186/s12903-022-02207-0

**Published:** 2022-05-13

**Authors:** Dejana Čolak, Alja Cmok Kučič, Tadeja Pintar, Boris Gašpirc, Rok Gašperšič

**Affiliations:** 1 Department of Oral Diseases and Periodontology, Dental Clinic, University Medical Centre Ljubljana, Hrvatski trg 6, 1000 Ljubljana, Slovenia; 2 Department of Abdominal Surgery, University Medical Centre, Ljubljana, Slovenia; 3 Faculty of Medicine, University of Ljubljana, Ljubljana, Slovenia

**Keywords:** Obesity, Bariatric surgery, Periodontist, Gingivitis, Hypertension, Cardiovascular risk factors

## Abstract

**Background:**

In obese patients, periodontitis might be associated with deprived systemic health. Edmonton obesity staging system (EOSS) is a new tool for classification of obesity that considers the metabolic, physical, and psychological health. The cross-sectional study aimed to evaluate the periodontal status of morbidly obese patients eligible for bariatric surgery and the association between periodontitis, obesity-related comorbidities, and EOSS.

**Methods:**

Morbidly obese patients eligible for bariatric surgery underwent detailed periodontal examination and were divided into the periodontitis group (PG) and the non-periodontitis group (NPG). The medical and demographic data were obtained from medical files, while behavioural data were obtained by the interview. Descriptive statistics and simple statistical tests were used to summarise the characteristics of the sample and the differences between PG and NPG. The logistic regression models were used to calculate the association (odds ratio (OR)) between periodontitis and obesity-related diseases and EOSS.

**Results:**

The study included 79 patients, with an average BMI of 44.6 kg/m^2^ (SD = 7.2). The prevalence of periodontitis was 65% (CI 95% 53%-75%). PG patients (n = 51) were older, more often smokers and were more often hypertensive than NPG patients (n = 28) (*p* < 0.05). Hypertension was positively associated with periodontitis with adjusted OR 3.98 (95% CI 1.23–12.8; *p* = 0.021)) and age with adjusted OR 1.06, (95% CI 1.01–1.13; *p* = 0.038)), while other tested conditions (diabetes, dyslipidaemia, and smoking habits) did not show significant association with periodontitis. Periodontitis did not correlate with EOSS or other obesity-related comorbidities (*p* > 0.05).

**Conclusion:**

The morbidly obese patients eligible for bariatric surgery show a high prevalence of periodontitis and, therefore, are advised to be examined by a dentist before undergoing surgery. They have higher odds of hypertension but not of other obesity-related diseases or higher stages of EOSS. The medical personnel should raise awareness among obese patients on the potential association of poor periodontal health with hypertension.

***Trial registration*:**

NCT04653714.

**Supplementary Information:**

The online version contains supplementary material available at 10.1186/s12903-022-02207-0.

## Introduction

Obesity is a growing epidemic associated with many systemic health sequels and a shortened life expectancy [1]. Edmonton obesity staging system (EOSS) is a new tool for determining obesity-related health problems and predictors of mortality [2]. EOSS is a five-stage system (0–4) that incorporates comorbidities, mental health, wellbeing, and functional limitations [3]. It provides more information on obesity-related health burdens than anthropometric measures (e.g., Body mass index (BMI), waist circumference) [4]. Bariatric surgery (BS) procedures are effective methods for the treatment of morbid obesity (BMI > 40 kg/m^2^) and obesity-related diseases [5]. Different BS interventions are founded on restriction, primary malabsorption, or a combination of restriction and malabsorption. In addition, the adoption of nonsurgical methods, i.e., lifestyle intervention and adaptive nutrition, are mandatory for the prevention of short-and long-term complications of BS [6].

Besides systemic health, obesity affects oral and periodontal health. Obesity-induced proinflammatory cytokines secreted from the adipose tissue may modulate the host response, promoting periodontal degradation in obese patients [7, 8]. Furthermore, many other risk factors for periodontitis and dental caries are prevalent in obese patients, such as diabetes mellitus [9], an unbalanced diet high in fermentable carbohydrates [10], and eating disorders [11]. Even though systemic health parameters improve remarkably after BS, periodontal degradation may progress during recovery from BS [7, 12–18], presumably resulting from changed oral microbiota composition [19, 20], eating habits [21], and some other consequences of BS (e.g., malnutrition, osteoporosis, increased regurgitation) [12, 22, 23]. Similarly, evidence also points to the damaging effect of BS on other aspects of oral health i.e., the progression of caries lesions [24], tooth erosion [12] and hypersensitivity of the teeth [13]. Despite worsening oral and periodontal health during recovery from BS [15–18], existing guidelines do not advise dental screening for BS patients' care [6, 25]. Even though obese patients are considered a risk group for periodontitis, the importance of oral health in these patients is commonly overlooked due to a lack of comprehensive data and awareness among medical personnel. Available limited research material demonstrated a wide range of periodontitis prevalence in obese patients eligible for BS (between 45 and 70% [26, 27]), yet it is lacking data on stage and grade of periodontitis distribution according to the recent periodontitis AAP/EFP diagnosis criteria [28].

Recent literature relates periodontitis with many obesity-related diseases [29]: metabolic syndrome [30], hypertension [31], diabetes mellitus [32], dyslipidaemia [33, 34], depression [35], polycystic ovary syndrome (PCOS) [36], and liver pathology [37]. The pathophysiological pathways behind these associations merge in systemic inflammation [38], insulin resistance [39], endothelial dysfunction [40, 41], oxidative stress [42] and gut dysbiosis [43]. In addition, periodontitis, and obesity-related comorbidities [44, 45] share common risk factors, such as age [46, 47], gender, genetic factors [33], smoking [47, 48], short education [49], and diabetes [47, 50]. Besides, periodontitis seems to be independently associated with hypertension, as shown by two recent systematic reviews [51, 52]. However, high heterogeneity among the included studies may be attributed to different study designs [53, 54], case definition [53] and inability to detect periodontitis-hypertension association [54–56]. Furthermore, the systematic review by Martin-Cabezas et al. [51] indicates that common risk factors hamper assessing the actual nature of the periodontitis-hypertension association. On the contrary, previous attempts associated periodontitis with metabolic syndrome [57] yet failed to associate periodontitis with hypertension. [58] Nevertheless, Foratori‐Junior et al. [59] still detected a higher prevalence of hypertension in morbidly obese patients with periodontitis.

Our study aimed to assess the prevalence and association of periodontitis, diagnosed by the new criteria (AAP/EFP classification) [28] and the above-mentioned systemic conditions (hypertension, metabolic syndrome, dyslipidaemia, diabetes, depression, and PCOS) in morbidly obese patients eligible for bariatric surgery (MOPEBS). As the correlation between periodontitis and EOSS has not been previously analysed, we hypothesize that MOPEBS with periodontitis will present with higher EOSS stages i.e., worse systemic health in comparison to non-periodontitis MOPEBS.

To sum up, in this cross-sectional study, we aimed to assess periodontal and systemic diagnostic parameters in MOPEBS and to analyse the potential association between periodontitis and obesity-related diseases and EOSS.

## Materials and methods

### Study design

In the cross-sectional study, MOPEBS underwent a detailed periodontal examination, while the diagnosis of obesity-related comorbidities was set by appropriate medical specialists. Patients were divided into the periodontitis group (PG) and non-periodontitis group (NPG) to compare their systemic health. The Strengthening the Reporting of Observational Studies (STROBE) guidelines were used to ensure the quality of the reporting in our cross-sectional study [60]. The study protocol was in accordance with the Declaration of Helsinki and was approved by the Republic Slovenia’s National Medical Ethics Committee (0120-312202010).

### Patient examination and data collection

All MOPEBS in the study were patients at the Department of Abdominal Surgery, University Medical Centre (UMC), Ljubljana, Slovenia, between January and December 2019 and March and September 2021. Only morbidly obese patients with the indication for BS, and without contraindication for the surgery, were referred to the dental clinic and consequently included in the study. The indication for the BS was made by the experienced bariatric surgeon (TP) following the published guidelines [6]. Patients were referred to a consultation with a bariatric surgeon by their primary care physician or other medical specialists, depending on the state of concomitant disease. BS was indicated if a patent had BMI > 40 or BMI > 35 and obesity-related comorbidity [6], and there were no other contraindications for general anaesthesia and the surgical procedure (e.g., severe CVD problems, medical and other general contraindication for surgery regarding the method of treatment and postoperative monitoring, previous multiple abdominal surgeries, medical history of malignant disease less than 5 years ago). After a short interview on periodontal health, dental pathology, and eating patterns, the bariatric surgeon referred the candidates for BS to the Department of Oral Medicine and Periodontology. All patients underwent a comprehensive dental and periodontal examination, performed by a single calibrated experienced examiner (ACK) blinded to patients' systemic health status. A calibration exercise for clinical parameters recession (REC) and probing pocket depth (PPD) (continues value) including 10 stage III/IV periodontitis patients yielded more than 95% of measurements within the 1 mm range. The intra-examiner kappa reliability values for diagnosis stages III/IV periodontitis diagnosis were over 0.90, while the intraclass correlation coefficient for PPD and REC was over 0.85. The number of teeth, fixed partial dentures, and removable partial dentures were recorded during a dental examination. The following periodontal parameters were recorded with a periodontal probe (POW6, Hu-Friedy, Chicago, Illinois, USA) on six sites of each existing tooth (mesiobuccal, buccal, distobuccal, mesiolingual, lingual, distolingual), excluding the third molars, to determine:

- the presence of dental plaque with Full-mouth Plaque Index (FMPI) [61],
- gingival inflammation with Full-Mouth Bleeding Score (FMBS) [62],
- bleeding on probing (BOP) (±),
- periodontal tissue destruction with probing pocket depth (PPD) (mm),
- gingival recession (REC) (mm) as the distance from cement-enamel junction to the gingival margin,
- tooth mobility (stage 0–3) [63], and
- furcation involvement (stage 0–3) [64].

Clinical attachment loss (CAL) was calculated post hoc from PPD and REC. If the peculiar non-periodontal reason for periodontal tissue destruction were suspected (e.g., endo-perio lesions, iatrogenic cause, orthodontic anomalies impacted 3rd molar distal to the 2nd molar, the gingival recession of traumatic origin, and dental caries in the cervical area), sites/teeth were excluded from the final evaluation.

The recorded periodontal parameters were used to set the proper periodontal diagnosis, assigned by the AAP/EFP classification [28]. To be categorised as a periodontitis case (PG), patients should exhibit detectable ≥ 1 mm interdental CAL on two or more nonadjacent teeth or buccal/oral CAL ≥ 3 mm with PPD of > 3 mm on two or more teeth. Periodontitis patients were further categorised by staging (I-IV) and grading system (A, B, C). Gingivitis was diagnosed if there were ≥ 10% sites with BOP, with PPD ≤ 3 mm, and without CAL or bone loss (NPG) [65]. Patients were considered periodontally healthy if there were < 10% of sites with BOP, PPD ≤ 3 mm, and without CAL or bone loss (NPG) [28].

The interview collected relevant patient information, such as demographic data (age, gender, level of education), reasons for a decision to undergo BS, information on behavioural habits such as smoking (no/less/more than 10 cigarettes per day), drinking alcohol (more/less than 12 alcohol units a month), weekly exercise (more/less than 3 times a week for at least 20 min), daily oral hygiene (using a toothbrush, fluoride toothpaste, and interdental hygiene tools), regular dental check-ups (at least twice a year), and the last periodontal therapy (more/less than 6 months ago).

Before undergoing dental examination, candidates for BS were evaluated for obesity-related diseases by medical specialists to determine the presence of these diseases, as suggested by the guidelines for BS [6]. For this purpose, the bariatric surgeon referred candidates for BS to a cardiologist, endocrinologist, pulmonologist, psychologist, orthopaedic surgeon, gynaecologist, and other specialists if needed, while esophagogastroduodenoscopy was performed by the coordinating bariatric surgeon (gastroenterologist). Data for the study were pulled from medical records at UMC. Diagnosis of metabolic syndrome, hypertension, dyslipidaemia, diabetes mellitus, obstructive sleep apnoea, polycystic ovary syndrome (PCOS), depression, orthopaedic disorders were noted. Diagnosis of hypertension (> 140/90 mmHg) was determined by a cardiologist based on the initial examination, repeated scintigraphy monument, ultrasound, Holter monitoring, and ambulatory blood pressure monitoring while accounting for previous hypertension diagnosis, and antihypertensive medication prescribed [50]. An endocrinologist made the current diagnosis of diabetes, dyslipidaemia, and PCOS (which was further explored by a gynaecologist if needed), based on clinical examination, biochemical blood analysis (diabetes: fasting blood glucose > 7 mmol/l or HbA1c > 6.5% or oral glucose tolerance test > 11.1 mmol/l or on medication for diabetes [66]; dyslipidaemia: triglyceride levels triglycerides > 1.7 mmol/l, HDL < 1 mmol/l; LDL > 3.4 mmol/l; or on medication for dyslipidaemia [67]); PCOS: oligo- or anovulation, biochemical hyperandrogenism (elevated total testosterone, or dehydroepiandrosterone sulphate, or androstenedione) or clinical hyperandrogenism or on medication for PCOS, while gynaecologist was consulted if needed (e.g., ultrasound to assess polycystic ovary morphology) [68]. The pulmonologist at the centres for sleeping disorders checked for the presence of undiagnosed obesity-related hypoventilation, obstructive sleep apnoea based on examination, symptoms and with nocturnal polysomnography, accounting for the previous diagnosis of obstructive sleep apnoea and the current use of continuous positive airway pressure (CPAP) masks. Using the standard diagnostic tool for person-centred therapy in candidates for BS, psychologists drew attention to possible psychological risk factors for poor treatment outcomes and made the diagnosis of depression or accounted for it if it was a previously made diagnosis. Patients were referred to an orthopaedic surgeon if there were signs and symptoms of orthopaedic disorders (osteoarthritis, rheumatoid arthritis, previous injury to the locomotor system due to excess weight, current pain in the locomotor system, and difficulty in mobility) [69]. All other diseases diagnosed were reported together as other diseases. Metabolic syndrome was diagnosed in obese patients if they fulfilled three of five following criteria pulled from medical records at UMC: (1) the waist circumference more prominent than 85 and 94 cm for women and men, respectively; (2) hypertension, or on medication for high blood pressure; (3) hypertriglyceridemia or on medication for it; (4) low HDL cholesterol: men: < 1.0 mmol/l, women: < 1.3 mmol/l, or on medication [70]. All specialists checked for the presence of contraindications for BS. At the consultation with the bariatric surgeon, weight in kg, height and waist circumference were measured in cm. Body mass index (BMI, kg/m^2^) was calculated from height and weight data [71]. Edmonton Obesity Staging System (EOSS) was determined by data available from the patients’ medical records [2]. Clinical and functional descriptions of the staging of obesity proposed by the authors Sharma et al. [72] were followed. Stages were set by determining obesity-related comorbidities, physical, psychological symptoms, impaired wellbeing, and functional limitations [72]. Briefly, EOSS stage 0 had no signs of the negative impact of obesity; stage 1 had borderline obesity-related risk factors and mild signs of obesity negative impact. Stages 2, 3 and 4 have established obesity-related comorbidities, with signs of physical, psychological symptoms, impaired wellbeing, and functional limitations due to obesity, ranging from moderate in stage 2 to severe in stage 4 [72].

### Participant inclusion and exclusion criteria

Including criteria were: ≥ 18 years and indication for any type of BS by the following criteria: BMI > 40 kg/m^2^, BMI ≥ 35 kg/m^2^ with at least one severe obesity-related comorbidities, based on current guidelines [6]. In addition, excluded were edentulous patients based on a clinical dental examination, pregnant or lactating female patients, patients with severe psychiatric disorders, and patients that refused to participate. Before the examination, all patients received a verbal explanation and signed written informed consent.

### Periodontitis group and non-periodontitis group in the study

Patients were divided into PG and NPG, based on their periodontal diagnosis. PG consisted of periodontitis patients regardless of periodontitis stage and grade. NPG patients consisted of gingivitis and periodontally health patients. Patients included in PG and NPG were patients from the same institution (UMC), indicated for BS by the same ordinating bariatric surgeon, examined by the same calibrated dental examiner without knowing patients ‘exact systemic condition.

### Sample size

For sample size calculation, a formula proposed by Charan et al. [73] for cross-sectional studies was applied. The available data on the prevalence of periodontitis in candidates for BS (70%) [74] was used, and with alpha set at 0.05 and margin absolute error at 10%, we estimate 80 participants are needed. The calculated sample size is more substantial than in the previously published studies on the same topic (n = 50) [74, 75].

### Statistical analysis

Descriptive statistics were used to summarise the characteristics of the sample, PG and NPG. If the comparison of numerical variables between the PG and NPG showed a tendency for normal distribution, the two-sample, two-sided t-test was applied, if not the Mann–Whitney U-test (two-tailed) was used. For categorical values, Fisher's exact test was used. To verify the association between periodontitis (outcome dichotomised 1, 0) and obesity-related diseases and EOSS, univariate logistic regression was used to calculate the odds ratio (OR) with a 95% confidence interval (95% CI). The outcome was dichotomised (1, 0) for hypertension, diabetes, metabolic syndrome, dyslipidaemia, PCOS, obstructive sleep apnoea, and depression, while EOSS was categorized in stages 0–4. If a parameter in univariate logistic regression showed a significant correlation with periodontitis at *p* < 0.25 [76–78], its association was further explored by multiple logistic models, including other independent covariates. For further correlation testing between EOSS and periodontitis, the ordinal logistic regression model was used, where the dependent variable was EOSS stage (0, 1, 2, 3, 4), and the explanatory variable was periodontitis (0, 1) and independent covariates. OR with 95% CI was therefore reported for all logistic regression models. Age (continuous: year), gender (1, female; 0, male), BMI (continuous: kg/m^2^), waist circumference (continuous: cm), smoking status ( ordinary: 0, non-smoker; 1, < 10 cigarettes a day; 2, > 10 cigarettes per day), alcohol consumption (dichotomized 0, < 12 units a month, 1 > 12 units a month), and physical activity (dichotomized 1, yes; 0, no), diabetes (dichotomized 1, yes; 0, no), dyslipidaemia (dichotomized 1, yes; 0, no), metabolic syndrome (dichotomized 1, yes; 0, no) were included in the multiple logistic regression analysis as confounding variable, when *p* < 0.25 was obtained by a univariate model [77]. The correlation between periodontal and dental parameters (continuous values: CAL, PPD, PPD > 4 mm, BOP, REC, number of teeth missing) and obesity-related diseases and EOSS was tested with logistic regression. The maximum number of independent variables in the multiple logistic regression model was set by the rule of 15 subjects per independent variable, nesting at 75 subjects being suitable for a maximum of 5 variables [59, 76]. Alpha was set at 0.05. Analysis was conducted in R (R Core Team 2020) [79], and Microsoft Excel [80].

## Results

**Fig. 1:**
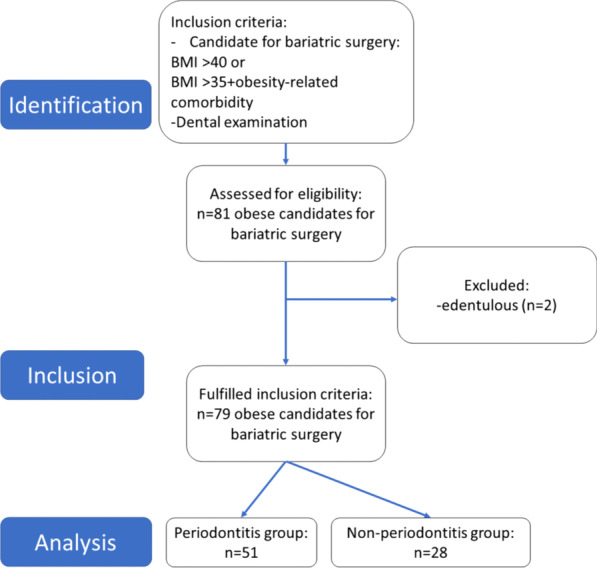
Flowchart of the patient inclusion process

The patient inclusion process is shown in the flow chart in Fig. 1.

The final sample consisted of 79 candidates for BS. The mean age of the sample was 47.2 years (SD = 12.6), the mean BMI was 44.6 kg/m2 (SD = 7.2), and there was a higher prevalence of female patients (around 70%). The prevalence of periodontitis in MOPEBS was high (63% (CI 95%, 52–74%)). From the sample, 51 patients (n = 51) were diagnosed with periodontitis and included in the PG. The remaining 28 patients (n = 28) with gingivitis (n = 25) or healthy periodontium (n = 3) were included in the NPG (Table 1).

**Table 1:** Periodontal diagnoses, staging and grading of periodontitis

Periodontal diagnoses	Count	Prevalence (95% CI)
Periodontitis	51	65% (53–75%)
Gingivitis	25	32% (22–44%)
Healthy	3	4% (1–11%)

Periodontitis stage			Periodontitis grade			Periodontitis extent		
Stage	Count	Prevalence (%)	Grade	Count	Prevalence (%)	Extent	Count	Prevalence (%)
I	9	18	A	12	24	l	12	24
II	12	24	B	16	30	g	38	74
III	19	36	C	23	46	m/i	1	2
IV	11	22						

*95% CI* 95% confidence interval; *l* localised (< 30% of teeth involved); *g* generalized (> 30% of teeth involved); *m/i* molar/incisor pattern

### Differences between morbidly obese patients with and without periodontitis

Patients in the PG were older than the NPG (P: 50.2 years, SD = 11.1; NP: 41.1 years, SD = 8.9, *p* = 0.00014). There was no difference between PG and NPG in education, alcohol consumption, weekly exercise, and the motivation for undergoing BS. However, PG had more heavy smokers (*p* = 0.0195). Patients in PG and NPG mainly stated they had inadequate daily oral hygiene and often missed the regular dental check-ups. The majority of the patients in PG and NPG confirmed they did not have any periodontal treatment, including nonsurgical periodontal therapy in the last 6 months (Table 2).

**Table 2:** Demographic and behavioral data of the sample and the comparison between periodontitis and non-periodontitis groups

Parameter	Data	All patients (n = 79)	Periodontitis group (n = 51)	Non-periodontitis group (n = 28)	Periodontitis vs non-periodontitis groups; *p* value
Gender (% of patients)	Female	73%	70%	78%	0.595^†^
	Male	27%	30%	28%	
Age (years)	Mean	46.9	50.2	41.1	0.000229**^,‡^
	SD	11.1	11.1	8.9	
	95%CI	44.3–49.4	47.1–53.4	37.3–44.6	
Education	Undergraduate	66%	67%	64%	1^†^
	Graduate	34%	33%	36%	
Smoking (per day)	None	67%	64%	71%	0.0195*^,^^†^
	< 10 cig	20%	16%	29%	
	> 10 cig	13%	20%	0%	
Alcohol consummation (per month)	< 12 unites	89%	84%	96%	0.148^†^
	> 12 unites	11%	16%	4%	
Weekly exercise	Yes	37%	31%	46%	0.225^†^
Daily oral hygiene	Yes	28%	25%	32%	0.603^†^
Regular dental check-ups	Yes	59%	55%	68%	0.339^†^
Last periodontal therapy	< 6 months	1%	0%	1%	1^†^
	> 6 months	99%	100%	99%	
Reason for BS	To lose weight	53%	38%	61%	0.098^†^
	To improve general health	47%	62%	39%	

*SD* standard deviation, *95% CI* 95% confidence interval

*p < 0.05; **p < 0.001; ^†^Fisher exact test; ^‡^*t* test

### Periodontal parameters

Periodontitis staging and grading are shown in Table 1. The most frequent stage was stage III (36%) and the most frequent grade C (46%). The PG had higher number of missing teeth (*p* < 0.001), presence of dentures (*p* < 0.001) in addition to worse FMPI (*p* < 0.001), CAL (*p* < 0.001), PPD (*p* < 0.001), REC (*p* < 0.001), furcation involvement (*p* < 0.01) and tooth mobility (*p* < 0.01) than NPG. There was no statistical difference between the two groups in BOP, FMBS, number of crowns, and pontic numbers (*p* > 0.05, Additional file 1: Table 1).

### Obesity-related diseases and parameters

PG and NPG did not differ in obesity-related parameters (waist circumference, BMI and EOSS; *p* > 0.05; Table 3). However, the PG showed a significantly higher prevalence of hypertensive patients than NPG (PG: 73%, NPG: 36%; *p* = 0.0019; Table 3). The prevalence of other obesity-related diseases is reported in Table 3. There was no difference in other obesity-related comorbidities (diabetes, metabolic syndrome, dyslipidaemia, PCOS, obstructive sleep apnoea, depression, and orthopaedic disorders) between PG and NPG (*p* > 0.05; Table 3). Hypertensive medication was similar between PG and NPG (*p* > 0.05) (Additional file 2: Table 2).

**Table 3:** Anthropometric data, obesity indexes, and prevalence of obesity-related diseases in the total sample, and the comparison between periodontitis and non-periodontitis groups

Parameter	Data	All patients (N = 79)	Periodontitis group (N = 51)	Non-periodontitis group (N = 28)	Periodontitis vs non-periodontitis groups; *p* value
Waist circumference (cm)	Mean	130.2	130.9	128.9	0.41^†^
	SD	16.2	15	17	
BMI (kg/m^2^)	Mean	44.6	44.5	44.5	0.96^†^
	SD	7.2	6.7	9	
	BMI > 40	71%	71%	71%	1^‡^
	BMI 35–39.9	29%	29%	29%	
EOSS stage (% of patients)	0	1%	0%	3.5%	0.228^‡^
	1	6%	4%	11%	
	2	44%	45%	43%	
	3	44%	49%	35.5%	
	4	4%	2%	7%	

Disease	All patients prevalence (95% CI)	Periodontitis group prevalence (95% CI)	Non-periodontitis group prevalence (95% CI)	Periodontitis vs non-periodontitis groups; *p* value
Diabetes mellitus	35% (24–46)	35% (22–50)	32% (16–52)	0.809^‡^
Hypercholesteremia	31% (21–42)	33% (21–48)	29% (13–49)	0.801^‡^
Hypertriglyceridemia	20% (12–31)	16% (7–29)	25% (11–45)	0.373^‡^
Dyslipidaemia	35% (25–47)	35% (22–50)	36% (19–56)	0.82^‡^
Hypertension	60% (49–71)	73% (58–84)	36% (19–56)	0.00193^*‡^
Obstructive sleep apnoea	44% (33–56)	47% (33–62)	39% (22–59)	0.63‡
Depression	17% (10–27)	18% (8–31)	18% (6–37)	1^‡^
Orthopaedic disorders	69% (58–79)	75% (60–86)	61% (41–78)	0.213^‡^
PCOS (% of females)	15% (5–20)	11% (2–19)	23% (6–37)	0.384^‡^
Other diseases	62% (50–72)	68% (54–81)	50% (31–69)	0.145^‡^
Metabolic syndrome	43% (32–55)	45% (31–60)	39% (22–59)	0.643^‡^

*BMI* body mass index, *EOSS* Edmonton Obesity Staging System, *PCOS* polycystic ovary syndrome

*p < 0.01; ^†^*t* test; ^‡^Fisher exact test

### The association between periodontitis and obesity-related diseases and EOSS

Multiple logistic regression models were used to explore further the association between periodontitis with hypertension, PCOS (in females) and EOSS, as they showed significance in the univariate logistic regression model. On the other hand, metabolic syndrome, dyslipidaemia, obstructive sleep apnoea, and depression did not show a tendency for correlation with periodontitis in the univariate logistic regression model and were not further explored with multiple logistic regression models.

In the final multiple regression model for the outcome hypertension, independent variables periodontitis, age, diabetes, smoking habits, and dyslipidaemia were included. The model revealed the presence of periodontitis (adjusted OR = 3.67, 95% CI 1.17–11.52; *p* = 0.0256) and higher age (adjusted, OR = 1.07, 95% CI 1.01–1.13; *p* = 0.0253) were positively associated with the diagnosis of hypertension (Table 4).

**Table 4:** Association between hypertension and independent variables in morbidly obese candidates for bariatric surgery (n = 79)

	Variables	OR	95% CI	*p*
Dependent value—hypertension				
Adjusted OR^†^	Periodontitis	3.98	1.23–12.8	0.021*
	Age	1.06	1.01–1.13	0.038*
	Smoking	0.86	0.4–1.87	0.71
	Diabetes	1.26	0.39–4	0.68
	Dyslipidaemia	1.88	0.61–5.7	0.27

*OR* odds ratio, *95% CI* 95% confidence interval

*p < 0.05; ^†^Multiple logistic regression model for the outcome hypertension and independent variables

The final multiple regression model for PCOS, which included periodontitis, age, and diabetes, revealed that presence of diabetes (adjusted OR = 10.16, 95% CI 1.63–63.19; *p* = 0.0128) and higher age (adjusted OR = 0.89, 95% CI 0.8–0.98; *p* = 0.027) was associated with PCOS.

In the ordinal logistic regression model for the outcome EOSS, periodontitis and age were included as independent variables. The model showed higher age (OR = 1.06, 95% CI 1.02–1.11; *p* = 0.0064) to be significantly associated with EOSS, while periodontitis was not (OR = 1.35, 95% CI 0.5–3.73; *p* = 0.55).

Dental and periodontal parameters (CAL, PPD, PPD > 4 mm, BOP, REC, number of teeth missing) were tested for potential correlation with obesity-related diseases and EOSS, but the significant associations were not detected (*p* < 0.05; data not shown).

## Discussion

In our cross-sectional study, periodontal parameters and obesity-related condition were recorded in MOPEBS. The prevalence of periodontitis in MOPEBS was higher than expected for the general population [81] and similar to the other BS populations [27]. In addition, the staging and grading system of periodontitis showed that MOPEBS presented with high severity and complexity scores and a high number of affected teeth. Another important finding is the extremely low prevalence of periodontally healthy patients, as most NPG patients were diagnosed with gingivitis. We can attribute this to the high dental plaque levels and presence of calcium channel blockers combined with hyperinflammatory state [8], high-frequency intake of refined carbohydrates [10], and eating disorders [11], all common in obese patients. These results point to mandatory periodontal intervention in these patients before BS, especially if we consider a possible increase in risk for periodontitis during recovery from BS [15].

A further finding of our study shows a positive association between periodontitis and hypertension in MOPEBS despite controlling for confounding factors. However, we did not find a higher prevalence of other obesity-related comorbidities and higher EOSS in MOPEBS with periodontitis. Even though the obesity-related comorbidities existed in MOPEBS with periodontitis, we speculate that the inability to detect the other periodontitis-obesity-related comorbidities association may result from the influence of age [82, 83]. Further exploration of these findings is needed on a more extensive, age balanced sample of morbidly obese patients. The significant periodontitis-hypertension association is apparent as both PG and NPG were balanced in most of the known confounding factors (e.g., obesity and obesity-related parameters, metabolic syndrome, diabetes mellitus, education level, daily oral hygiene, alcohol consumption) [50, 84]. Regarding obesity and the periodontitis-hypertension association, most previous studies with heterogeneous populations adjusted for BMI/obesity [85–89] or rarely excluded patients with obesity [90], while not many studies explored this association in only obese patients. In addition, the difference in age and smoking habits between PG and NPG was addressed by including them in the final model. Results show that age was also associated with hypertension, yet the periodontitis-hypertension relationships remained significant even after including age in the model.

These findings align with the study by Foratori‐Junior et al. [59], who also found a positive association between hypertension and periodontitis and hypertension and age in candidates for BS. In comparison to our study, Foratori‐Junior et al. [59] applied more stringent inclusion criteria, excluding smokers, patients with diabetes, and calcium channel blockers to reduce the possible effects of these confounders. In contrast to the above study, PG and NPG in our study were better balanced regarding education level and other behaviour patterns or factors that may influence periodontitis and hypertension [50, 84, 91]. The findings of our and the previously mentioned study [59], suggest the odds of hypertension in morbidly obese patients with periodontitis might be higher than in subjects with periodontitis and lower BMI [56, 92]. The previous meta-analysis of the studies that mostly adjusted for obesity, found diagnoses of moderate-severe periodontitis associated with hypertension with OR = 1.22 [52]. On the other hand, our study on morbidly obese patients associated periodontitis and hypertension with an even higher odds ratio (OR = 3.98), pointing to the potentially more significant impact of periodontitis on cardiovascular diseases in a morbidly obese population.

Several explanations were proposed in the literature to explain the nature of the periodontitis-hypertension association [31]. Firstly, hypertension might negatively influence the microcirculation of periodontal tissue [52, 93]. Second, as suggested in the study by Khocht et al. [88], hypertension may lead to high inflammatory markers in healthy/non-inflamed periodontal sites promoting periodontitis onset. Finally, another potential indirect hypertension pathway is through antihypertensive medication, e.g., calcium channel blockers, linked to gingival overgrowth and pseudo pockets formation [91]. On the other hand, there is a body of evidence suggesting that periodontitis might pose a predominantly negative influence over hypertension-related pathways: periodontitis has been shown to increase systemic inflammation, oxidative stress, endothelial dysfunction, atherosclerosis, as well as to increase insulin resistance, promote dyslipidaemia and liver disease, thereby directly or indirectly influencing hypertension [94–96]. Thus, the findings of our study support the presence of periodontitis-hypertension association, yet, without determining the direction of the association.

This is the first study to explore the potential association between EOSS and periodontitis to the best of our knowledge. We could not show an association with periodontitis, while age was positively correlated to EOSS. Still, exploring periodontitis-EOSS association and association between EOSS and other oral health parameters on the larger sample should be considered in future studies.

The limitation of our study was a relatively small sample size. Nevertheless, our sample was considered sufficient to describe the periodontal and systemic health condition of MOPEBS and explore periodontitis-obesity-related comorbidities relation. Furthermore, as age is a confounding factor [47, 82, 83, 97], better-matched cases and controls would give us more substantial evidence for hypertension-periodontitis and periodontitis-obesity-related comorbidities association. Finally, as BS patients often had diabetes mellitus, diabetes may influence periodontitis and hypertension [47, 50]. Our sample, however, showed no difference in diabetes mellitus prevalence between PG and NPG.

In conclusion, the results of our study indicate that in morbidly obese eligible for BS the prevalence of periodontitis is high, and hypertension is positively associated with periodontitis, regardless of confounding factors. Other obesity-related comorbidities and EOSS, despite being present in morbidly obese eligible for BS, do not correlate with periodontitis. Nevertheless, dental professionals should examine candidates for BS before undergoing BS as periodontitis may influence their systemic health, in particular hypertension. Future studies on the effects of periodontitis on obesity-related comorbidities and EOSS in obese patients are anticipated.

## Supplementary Information

**Additional file 1.** **Supplemental Table 1.** Periodontal parameters of the sample and the comparison between periodontitis and non-periodontitis groups.

**Additional file 2.** **Supplement Table 2.** Medication intake in patients with hypertension (n = 47).

## Data Availability

The datasets used and analysed during the current study available from the corresponding author on reasonable request. Parts of the data from the study represent the content of the thesis of DČ.

## Abbreviations

MOPEBS: Morbidly obese patients eligible for bariatric surgery
EOSS: Edmonton obesity staging system
BMI: Body mass index
BS: Bariatric surgery
CVD: Cardiovascular diseases
CANDIDATES FOR BS: Obese patients indicated for bariatric surgery
FMPI: Full-mouth Plaque Index
FMBS: Full-Mouth Bleeding Score
BOP: Bleeding on probing
PPD: Probing pocket depth
CEJ: Distance from cement-enamel junction to gingival border
REC: Gingival recession
CAL: Clinical attachment loss
PG: Periodontitis group
NPG: Non-periodontitis group
SD: Standard deviation
OR: Odds ratio
95% CI: 95% confidence interval
UMC: University Medical Centre
