# Supplementary material for: Periodontal and systemic health of morbidly obese patients eligible for bariatric surgery: a cross-sectional study
Source: BMC Oral Health. 2022 May 13;22:174. doi: 10.1186/s12903-022-02207-0 (PMC9107195; doi:10.1186/s12903-022-02207-0)
Supplement: Supplementary file 1 — Additional file 1. Supplemental Table 1. Periodontal parameters of the sample and the comparison between periodontitis and non-periodontitis groups. [file 12903_2022_2207_MOESM1_ESM.docx]

**Supplemental table 1:** Periodontal parameters of the sample and the comparison between periodontitis and non-periodontitis groups

| Periodontal parameter | Data | Total sample  N=79 | Periodontitis group  N=51 | Non-periodontitis group  N=28 | p value |
| --- | --- | --- | --- | --- | --- |
| Missing teeth  (number) | 25^th^ | 2 | 3 | 0.5 | 0.00004 ** † |
|  | 50^th^ | 4 | 7 | 2 |  |
|  | 75^th^ | 10 | 12 | 5.5 |  |
| BOP  (% of sites) | 25^th^ | 14 | 17.3 | 12.5 | 0.287 ‡ |
|  | 50^th^ | 26 | 27 | 19 |  |
|  | 75^th^ | 41 | 43.5 | 32 |  |
| FMBS  (% of sites) | 25^th^ | 2.9 | 2.8 | 2.5 | 0.138 † |
|  | 50^th^ | 6.5 | 10.3 | 5 |  |
|  | 75^th^ | 16.8 | 19.5 | 9.6 |  |
| FMPI  (% of sites) | 25^th^ | 28.5 | 38.8 | 21 | 0.00033 * ‡ |
|  | 50^th^ | 47 | 51 | 29 |  |
|  | 75^th^ | 69 | 74.8 | 48.5 |  |
| PPD  (mm) | 25^th^ | 2.4 | 2.5 | 2.3 | 0.00000 ** ‡ |
|  | 50^th^ | 2.7 | 2.9 | 2.4 |  |
|  | 75^th^ | 3 | 3.2 | 2.4 |  |
| PPD>4mm  (% of sites) | 25^th^ | 1.5 | 8.9 | 0 | 0.00000 ** † |
|  | 50^th^ | 8 | 16.5 | 1.5 |  |
|  | 75^th^ | 22 | 29.1 | 2.7 |  |
| CAL  (mm) | 25^th^ | 0.1 | 0.6 | 0 | 0.00000 ** † |
|  | 50^th^ | 0.6 | 1.2 | 0.1 |  |
|  | 75^th^ | 1.6 | 2.3 | 0.1 |  |
| REC  (mm) | 25^th^ | 1 | 1 | 0 | 0.00000 ** † |
|  | 50^th^ | 1.2 | 1.5 | 0.6 |  |
|  | 75^th^ | 1.8 | 2 | 1.2 |  |
| REC ≥1mm  (% of sites) | 25^th^ | 1 | 4.9 | 0 | 0.000004  ** † |
|  | 50^th^ | 8 | 18.5 | 2 |  |
|  | 75^th^ | 27 | 34.8 | 5 |  |
| Furcation | 25^th^ | 0 | 0 | 0 | 0.00962 * † |
|  | 50^th^ | 0 | 0 | 0 |  |
|  | 75^th^ | 1 | 1 | 0 |  |
| Furcation  (% of sites) | 25^th^ | 0 | 0 | 0 | 0.0079  * † |
|  | 50^th^ | 0 | 0 | 0 |  |
|  | 75^th^ | 8 | 24.6 | 0 |  |
| Tooth mobility | 25^th^ | 0 | 0 | 0 | 0.00565  * † |
|  | 50^th^ | 0 | 0 | 0 |  |
|  | 75^th^ | 1 | 1 | 0 |  |
| Tooth mobility  (% of teeth) | 25^th^ | 0 | 0 | 0 | 0.0034  * † |
|  | 50^th^ | 0 | 4 | 0 |  |
|  | 75^th^ | 7.5 | 15 | 0 |  |
| Crowns  (number) | 25^th^ | 0 | 0 | 0 | 0.0702 † |
|  | 50^th^ | 0 | 1 | 0 |  |
|  | 75^th^ | 4 | 5 | 1 |  |
| Pontic  (number) | 25^th^ | 0 | 0 | 0 | 0.099 † |
|  | 50^th^ | 0 | 0 | 0 |  |
|  | 75^th^ | 1 | 1.8 | 0 |  |
| Dentures  (% of patients) | no dentures | 86% | 78% | 100% | 0.00651 * § |
|  | upper denture | 9% | 14% | 0% |  |
|  | lower denture | 0% | 0% | 0% |  |
|  | both dentures | 5% | 8% | 0% |  |

*Abbreviations: SD: standard deviation; 25^th^: 25^th^ percentiles; 50^th^: median; 75^th^: 75^th^ percentiles; PPD: probing pocket depth; REC: gingival recession; BOP: bleeding on probing; CAL: clinical attachment loss; FMPI: Full-mouth Plaque Index; FMBS: Full-Mouth Bleeding Score; * p < 0.01; **p<0.001; †: Mann Witney test; §: Fisher exact test.*
