# Supplementary material for: Periodontal and systemic health of morbidly obese patients eligible for bariatric surgery: a cross-sectional study
Source: BMC Oral Health. 2022 May 13;22:174. doi: 10.1186/s12903-022-02207-0 (PMC9107195; doi:10.1186/s12903-022-02207-0)
Supplement: Supplementary file 2 — Additional file 2. Supplement Table 2. Medication intake in patients with hypertension (n = 47). [file 12903_2022_2207_MOESM2_ESM.docx]

**Supplement table 2:** Medication intake in patients with hypertension (n=47)

| Type of medication | Periodontitis group with hypertension (n=37) | | Gingivitis group with hypertension (n=10) | | p value † |
| --- | --- | --- | --- | --- | --- |
|  | number of patients | % patients | number of patients | % patients |  |
| CA blockers | 10 | 25% | 4 | 44% | 0.45 |
| Beta-blockers | 14 | 39% | 3 | 22% | 1 |
| ACE inhibitor | 8 | 22% | 1 | 11% | 1 |
| Angiotensin II receptor blockers | 1 | 3% | 1 | 11% | 1 |
| Statin | 1 | 3% | 1 | 11% | 1 |
| Vasodilator | 2 | 6% | 0 | 0% | 1 |
| Diuretic | 4 | 11% | 0 | 0% | 1 |

*Abbreviations: †: Fishers exact test*
